# A Celecoxib-Loaded Emulsion Gel for Enhanced Drug Delivery and Prevention of Postoperative Adhesion

**DOI:** 10.3390/pharmaceutics17040427

**Published:** 2025-03-27

**Authors:** Heesang Yang, Dongmin Kim, Jong-Ju Lee, Ye Ji Kim, Seungeun Song, Sooho Yeo, Sung-Joo Hwang

**Affiliations:** College of Pharmacy, Yonsei Institute of Pharmaceutical Sciences, Yonsei University, 85 Songdogwahak-ro, Yeonsu-gu, Incheon 21983, Republic of Korea; hjyang2190@gmail.com (H.Y.); kimtz01@naver.com (D.K.); leejj8862@yonsei.ac.kr (J.-J.L.); 5_346@naver.com (Y.J.K.); tmddms6085@naver.com (S.S.)

**Keywords:** celecoxib, pectin, emulsion gel, anti-adhesion, non-steroidal anti-inflammatory drugs

## Abstract

**Background:** Postoperative adhesions are a common complication following abdominal surgery, affecting over 90% of patients and leading to significant morbidity. Current anti-adhesion strategies, such as the use of physical and chemical barriers, have limitations such as short retention time, mechanical fragility, and inefficient drug delivery. This study developed a pectin-based emulsion gel loaded with celecoxib to prevent adhesions and provide localized pain relief. **Methods**: Formulations (F1–F4) with different pectin concentrations were evaluated for rheological properties, mucoadhesion, degradation rate, and celecoxib release. In vivo efficacy was evaluated in Sprague−Dawley rats via a standardized model of peritoneal abrasion, in which the formulations were compared to a commercially available anti-adhesion barrier. **Results**: The optimized emulsion gel (F4) exhibited improved mucoadhesion (9009 mPa·s), prolonged retention, and controlled celecoxib release over 14 days, reaching 80% release by day 9. In vivo, formulation F4 significantly reduced adhesions compared to a commercially available product. Pharmacokinetic analysis showed rapid absorption (T_max_ = 2 h) and sustained celecoxib plasma levels, confirming its effectiveness as a localized drug-delivery system. The celecoxib-loaded pectin-based gel successfully prevented postoperative adhesions and provided sustained pain relief. **Conclusions**: These findings suggest its potential clinical utility, though further preclinical and clinical evaluations are required.

## 1. Introduction

Adhesion formation in the abdominal cavity is a common postoperative complication, occurring in more than 90% of patients after surgery [1]. While adhesion is a natural biological response during tissue injury and repair, it can negatively impact the function of adjacent organs or tissues [2]. In severe cases, adhesions may lead to life-threatening complications, often necessitating surgical intervention for removal. Adhesion formation occurs as part of the wound-healing process following tissue injury, during which the coagulation cascade is activated, leading to the production of fibrin. Fibrin is essential for normal tissue repair; however, excessive fibrin deposition promotes fibroblast proliferation and neovascularization, ultimately resulting in adhesion formation [3]. The coagulation process begins with the activation of prothrombin, which leads to the generation of thrombin. Thrombin then converts fibrinogen into fibrin, facilitating tissue repair and wound healing. Under normal conditions, fibrin undergoes fibrinolysis, in which inactive plasminogen is converted into active plasmin by tissue plasminogen activator (tPA) or urokinase plasminogen activator (uPA) [4]. However, when fibrin degradation is impaired, excessive fibrin accumulates, forming a fibrin matrix [5]. This matrix adheres to surrounding tissues, ultimately leading to the development of adhesions.

Several strategies have been explored to prevent postoperative adhesion, including the use of chemical anti-adhesion barriers and physical anti-adhesion barriers [3]. Chemical methods involve administering anti-inflammatory agents, fibrinolytic agents, anticoagulants, and antibiotics, all of which play a role in inhibiting adhesion formation [6]. Anti-adhesion barriers are commercially available in film, solution, and hydrogel formulations. These barriers function by physically covering the surgical site to prevent adhesions [2]. However, film-based barriers require skilled application techniques and are primarily suited for use in laparoscopic surgery due to their fragile mechanical properties [7]. Solution-based formulations are prone to rapid degradation, leading to detachment from the application site and limiting their anti-adhesion efficacy. Hydrogels, unlike these formulations, have a relatively long residence time at the application site and can be conveniently administered [8]. Pectin, a plant-derived polysaccharide, is widely utilized in the food and pharmaceutical industries due to its hydrophilic properties and structural composition, which includes ester and carboxylic acid groups [9]. It exhibits excellent biocompatibility and mucoadhesive properties, making it a valuable material for use as a gelling agent and stabilizer. Additionally, pectin has demonstrated anti-inflammatory, wound-healing, and anti-adhesion effects [10]. The mucoadhesive nature of pectin prolongs residence time at the application site, leading to enhancement of both the anti-adhesion effect and drug-delivery efficiency [11].

Postoperative pain management typically involves the administration of opioid analgesics and nonsteroidal anti-inflammatory drugs (NSAIDs) [12]. However, opioid analgesics pose significant risks, including respiratory depression and physical dependence, which can be dangerous for patients. In contrast, NSAIDs provide analgesic, antipyretic, and anti-inflammatory effects [13]. NSAIDs exert their effects by inhibiting cyclooxygenase (COX), thereby blocking the conversion of arachidonic acid to prostaglandins (PGs) [14]. COX-1 is expressed in the gastrointestinal mucosa, where it plays a crucial role in maintaining vascular homeostasis and platelet aggregation. Additionally, COX-2 is rapidly induced in response to inflammatory stimuli; this leads to the local production of PGs, which contribute to pain and inflammation [15,16]. Celecoxib (CEL) is a selective inhibitor of COX-2, offering the advantage of reducing pain and inflammation without affecting COX-1 function [17,18,19]. Therefore, the incorporation of CEL as a chemical agent into an anti-adhesion material has the potential to effectively inhibit adhesion formation while simultaneously managing postoperative pain.

We previously designed thermosensitive hydrogels [20] and polysaccharide-based emulsion gels [21] loaded with 5-fluorouracil, a hydrophilic drug, for both anti-adhesion and anti-cancer applications. Recently, research has been conducted on the use of emulsions as anti-adhesion agents. However, a major drawback of emulsions is their inability to form a solid physical barrier, which is crucial for effective adhesion prevention. To address this limitation, we investigated the incorporation of pectin, a polysaccharide, to enhance the anti-adhesion properties of emulsions. Pectin improves biocompatibility, biodegradability, viscoelasticity, and mucoadhesive properties, thereby increasing the swelling capacity of the product. This swelling characteristic compensates for the lack of a solid physical barrier, effectively overcoming the limitations of conventional emulsions in adhesion prevention [22]. In this study, we developed pectin-based emulsion gels containing CEL, a lipophilic drug, for use as an advanced anti-adhesion agent. In particular, the exclusive use of pectin resulted in a significant reduction in viscosity compared to the gels developed in our previous studies, allowing the emulsion gel to exhibit sol-like liquid characteristics. Thus, our CEL-loaded pectin-emulsion gels could be useful as a lipophilic drug-delivery system with anti-adhesion agents with improved spreadability and residence time.

## 2. Materials and Methods

### 2.1. Materials

CEL was provided by DaeHwa Pharmaceutical Co., Ltd. (Sungnam, Republic of Korea), and atorvastatin was obtained from DaeWoong Pharmaceutical Co., Ltd. (Seoul, Republic of Korea). Purified soybean oil was gifted by Lipoid GmbH (Ludwigshafen, Germany). Pectin powder (GENU^®^, citrus type USP/200) was obtained from CP Kelco (Atlanta, GA, USA). Polysorbate 80 (Montanox^®^ 80) was kindly provided by Seppic (Paris, France). Kolliphor^®^ P 407 (P 407) and Kollisolv^®^ PEG 400 (PEG 400) were purchased from BASF (Ludwigshafen, Germany). Mucin type II was acquired from Sigma Aldrich (St Louis, MO, USA). All other chemicals used were of reagent grade.

### 2.2. Preparation of CEL-Loaded Pectin-Emulsion Gel

For the preparation of the emulsion gel, P 407, Montanox^®^ 80, pectin, celecoxib, and PEG 400 were combined with distilled water and mixed under high-speed stirring to form the hydrogel phase. The hydrogel phase containing PEG and pectin was stirred at room temperature for 1.5 h to ensure proper hydration. After the hydration was complete, the hydrogel phase and soybean oil were prepared at 25 °C. Soybean oil was then slowly added dropwise into the hydrogel phase and dispersed using a T18 basic Ultra-Turrax^®^ Homogenizer (IKA, Staufen, Germany) at 9500 rpm for 10 min (Table 1).

A preliminary study tested a formulation with 9% pectin, but it was not selected due to its high viscosity, which limited syringeability and made it impractical for application. Based on these findings, pectin concentrations of 5% to 8% were chosen, as concentrations above 8% resulted in excessive viscosity, hindering both preparation and injection, while concentrations below 5% were expected to be insufficient for retention at the surgical site.

### 2.3. Rheological and Viscosity Analysis

Rheological properties were evaluated using an MCR 702 MultiDrive Rheometer (Anton Paar, Graz, Austria) equipped with a 25 mm parallel plate geometry (PP25). Dynamic viscoelasticity was measured by determining the strain value within the linear viscoelastic region (LVR) through amplitude sweep testing. An appropriate amount of emulsion was placed on the lower plate, and the gap between the plates was set to 1 mm. All measurements were conducted at 25 °C, with a frequency range of 0.1 to 10 rad/s. The shear strain was fixed at 0.5%.

Viscosity (η) curves were obtained over a range of shear rates (1/s) from 0.1 to 100. The measurement temperature was maintained at 25 °C, and the same equipment and setup as described above were used.

### 2.4. In Vitro Mucoadhesion Evaluation

The mucoadhesive test was performed using a slightly modified method [11,23]. Interactions with mucins present in the abdominal cavity indicate that the formulations are likely to adhere well to the mucosa [24]. Mucin suspensions were prepared at concentrations of 5% (*w*/*v*) and 10% (*w*/*v*) in phosphate-buffered saline (PBS) buffer (pH 7.4). The prepared 10% mucin suspension was mixed with the emulsions (F1, F2, F3, and F4) in equal-volume ratios to evaluate their interactions with mucin. Additionally, PBS buffer and emulsions were mixed at the same volume ratios using a spatula to ensure the diluted concentrations of the formulations in the mucin suspension were consistent. The measured viscosity (η) was calculated according to Equation (1), as follows:

η_mix_ = η_p_ + η_m_ + Δη (1)

η_mix_: viscosity of emulsion and 10% mucin suspension mixture,
η_p_: viscosity of emulsion diluted with PBS buffer,
η_m_: viscosity of 5% mucin suspension.

### 2.5. Evaluation of Particle Size and Zeta Potential 

Particle size and zeta potential were measured using dynamic light scattering (DLS) and electrophoretic light scattering (ELS) with a nanolaser particle-size analyzer (ELSZ-1000, Otsuka Electronics Co., Osaka, Japan). The emulsion was diluted 1000-fold in distilled water, and measurements were performed in triplicate for 25 s each. Scattered light was detected at a 90° angle.

### 2.6. In Vitro Evaluation of Emulsion-Gel Degradation 

The emulsion-gel-degradation study was conducted based on the method described, with slight modifications [21]. First, 1 g of the emulsion formulation was placed in a 50 mL conical tube. PBS buffer (pH 7.4), pre-warmed to 37 °C, was prepared, and 20 mL of the buffer was slowly added to the tube containing the emulsion, using a pipette to completely submerge it. The volume of PBS buffer was selected based on the average amount of peritoneal fluid in the human abdomen [25]. The conical tube was then placed in a water bath maintained at 37 °C. At predetermined time points, the tube was removed, the PBS buffer was discarded, and the remaining emulsion was weighed.

(2) $$\mathrm{Emulsion-gel weight}\;(\%)=\frac{\mathrm{Emulsion-gel weight at time point}}{\mathrm{Initial emulsion-gel weight}}\times 100$$

### 2.7. In Vitro CEL Release Test

An in vitro dissolution study was conducted to determine the release profile of CEL from the anti-adhesion emulsion gels. A dialysis bag containing 1 g of emulsion gel was placed in a 50 mL conical tube, after which 40 mL of pre-warmed PBS buffer (pH 7.4) containing 1% sodium lauryl sulfate (SLS) was added. The tube was then placed in a shaking water bath (HB-205SW, Hanbaek Scientific Company, Bucheon, Gyeonggi-do, Republic of Korea) set at 37 °C and 50 rpm. At predetermined time points, 1 mL of medium was withdrawn and replaced with an equal volume (40 mL) of fresh buffer to maintain sink conditions.

For the control group, a commercially available Celebrex^®^ capsule (100 mg, Viatris Co., Canonsburg, PA, USA) was used without employing a dialysis bag. The capsule contents were directly dispersed in 40 mL of PBS buffer (pH 7.4) containing 1% sodium lauryl sulfate (SLS) and subjected to the same shaking water bath conditions (37 °C, 50 rpm). At predetermined time points, 1 mL of the sample was withdrawn from the conical tube and replaced with an equal volume of fresh buffer (1 mL) to maintain sink conditions.

HPLC analysis was performed using an Agilent HPLC system (1200 Infinity Series, Agilent Technologies, Waldbronn, Germany). A C18 column (CAPCELL, 120 Å pore size, 5 µm, 4.6 mm inner diameter × 250 mm, Shiseido, Tokyo, Japan) was used for separation, and detection was conducted at 258 nm using an HPLC-UV spectrometer (Agilent 1290 Infinity, Agilent Technologies, Waldbronn, Germany). The mobile phase consisted of 0.2% trifluoroacetic acid (TFA) and acetonitrile (ACN) in a 30:70 volume ratio, with a flow rate of 1.5 mL/min.

### 2.8. In Vivo Evaluation of Intraperitoneal Anti-Adhesion Effects

Male SD rats (8 weeks old, weighing approximately 250 g) were obtained from Raon Bio Company (Yongin, Gyeonggi-do, Republic of Korea). The animals were acclimatized for 1 week under standard dietary and environmental conditions. A total of 36 rats were randomly assigned to six groups: control, Medicurtain^®^ (Shin Poong Pharm Co., Ltd., Ansan, Republic of Korea), F1, F2, F3, and F4, with six animals in each group.

Anesthesia was induced using a small-animal respiratory anesthesia machine with isoflurane (Hana Pharm. Co., Ltd., Pochon, Kyonggi-do, Republic of Korea). Following induction, a 5 cm midline incision was made to expose the abdominal cavity and the peritoneum was abraded 200 times with sandpaper (1 cm × 1 cm). The control group received no treatment, while 0.5 mL of the F1, F2, F3, F4, and Medicurtain^®^ product was applied directly to the wound site in the F1, F2, F3, F4, and Medicurtain^®^ groups, respectively. After treatment, the abdominal cavity was closed using 3-0 coated VICRYL^TM^ Polyglactin 910 sutures (J & J International, La Louvière, Belgium).

Ten days post-operation, a second abdominal incision was performed to assess adhesion severity. Adhesions were scored based on size, thickness, and strength using a 0-to-4 grading scale, as described in Table 2 [26].

### 2.9. Histopathological Evaluation of Intraperitoneal Tissue

For histopathological evaluation, tissue samples recovered from necropsy were fixed in 10% neutral buffered formalin, then subjected to dehydration, paraffin embedding, and sectioning into 5 µm thick slices. The sections were then stained with hematoxylin and eosin (H&E) using standard histological techniques to assess tissue morphology.

Tissue samples were collected from six experimental groups (control, Medicurtain^®^, F1, F2, F3, and F4), specifically from the site of induced peritoneal injury. In each group, samples were obtained from both rats with adhesion formation and those without adhesion formation. For rats without adhesion formation, tissue was excised from the same injury site where adhesion was initially induced. This approach allowed for a comparative histopathological evaluation between tissues with and without adhesion formation at the same injury site.

### 2.10. In Vivo Pharmacokinetics (PK) Test

Male rats (8 weeks old, weighing approximately 250 g) were purchased from Raon Bio Company (Yongin, Gyeonggi-do, Republic of Korea). Animals were acclimatized for 1 week before the experiment under standard diet and environmental conditions in compliance with the IACUC guidelines. A total of 12 rats were divided into 4 groups (F1, F2, F3, and F4), with 3 rats in each group. The administration procedure followed the method described in Section 2.8, and each group was treated with formulations containing CEL. The dose administered was equivalent to a CEL concentration of 6.45 mg/kg.

Blood samples (0.2 mL) were collected via the tail vein at predetermined time points. Samples were centrifuged at 13,000 rpm for 10 min to separate plasma, which was then stored at −80 °C. For analysis, 50 μL of plasma was mixed with 50 μL of methanol and 900 μL of internal standard (IS) solution (atorvastatin) in a 1.5 mL Eppendorf^®^ tube (Sigma Aldrich, St Louis, MO, USA). The mixture was vortexed for 30 s and centrifuged at 15,000 rpm for 15 min. The supernatant was filtered using a 0.2 μm PTFE syringe filter.

Analysis was performed using an Agilent 6490 Triple Quadrupole LC/MS system equipped with a Waters Xbridge BEH C18 column (particle size 3.5 μm, 2.1 mm × 30 mm, Waters, Eschborn, Germany). The mobile phase consisted of 0.1% formic acid in water and acetonitrile (ACN) in a 60:40 volume ratio. The injection volume was 3 μL, with a flow rate of 1 mL/min and a total run time of 3 min. Multiple reaction monitoring (MRM) transitions were set as follows: CEL (380 → 315.9, collision energy: −30 eV) and atorvastatin (559.2 → 440, collision energy: 20 eV). Data acquisition and processing were performed using Agilent 1290 Infinity MassHunter Qualitative and Quantitative Analysis software.

## 3. Results and Discussion

### 3.1. Rheology and Viscosity of Emulsion Gels

The viscoelasticities of the emulsion gels were evaluated using frequency sweep measurements to obtain insight into their structural integrity and rheological properties. The storage modulus (G′) and loss modulus (G″) were measured across a range of angular frequencies to assess the viscoelastic behavior of each formulation. G′ represents the elastic (solid-like) component of the material and indicates energy storage during deformation, while G″ reflects the viscous (liquid-like) component and represents energy dissipation as heat [27].

**Figure 1:**
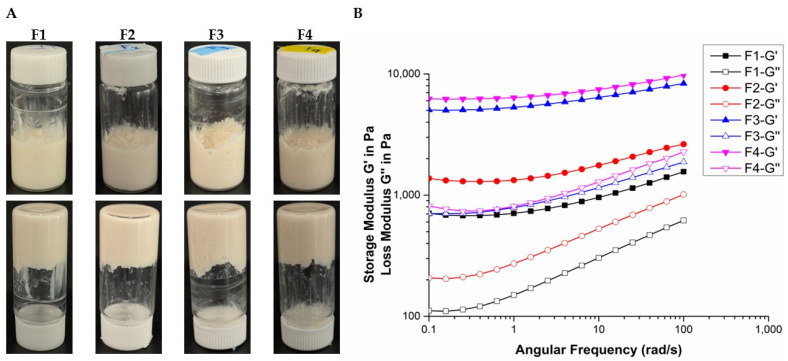
(**A**) Appearance of CEL-loaded emulsion gels. (**B**) Dynamical oscillatory frequency sweep test curves of emulsion gels. Open symbols represent loss modulus and filled symbols represent storage modulus. F1: pectin 5%, F2: pectin 6%, F3: pectin 7%, and F4: pectin 8%.

The storage modulus G′ was consistently higher than G″ across the entire frequency range for all formulations, confirming the formation of a stable network structure (Figure 1). This stability is attributed to the dominance of elastic behavior over viscous behavior [28,29]. At a low angular frequency of 0.1 rad/s, the value of G′ was significantly larger than that of G″, suggesting excellent dispersion stability. This characteristic is advantageous for long-term storage, as it prevents phase separation and structural breakdown. Furthermore, from a rheological perspective, the formulations exhibit shear-thinning behavior, indicating that the gel can be easily spread during application while maintaining structural stability post-application. This property enhances usability and retention, making the formulation more effective in practical applications. To further investigate the viscoelastic properties, the loss tangent (tan⁡δ), defined as the ratio of G″ to G′ (tan⁡δ=G″/G′), was analyzed. The loss tangent provides a direct comparison of the viscous and elastic properties of the formulations. A smaller loss tangent, closer to 0°, indicates predominantly elastic behavior, whereas a larger tangent, approaching 90°, signifies stronger viscous properties.

At a higher angular frequency of 10 rad/s, notable differences in viscoelastic behavior were observed among the formulations (Table 3). Formulation F1 exhibited the largest loss-tangent angle, indicating that it had the most pronounced viscous properties and more liquid-like behavior. In contrast, F4 had the smallest loss-tangent angle, reflecting the most pronounced elastic properties and a more solid-like network structure. These results highlight the distinct structural characteristics of each formulation and their potential suitability for specific applications requiring either elastic or viscous dominance.

**Figure 2:**
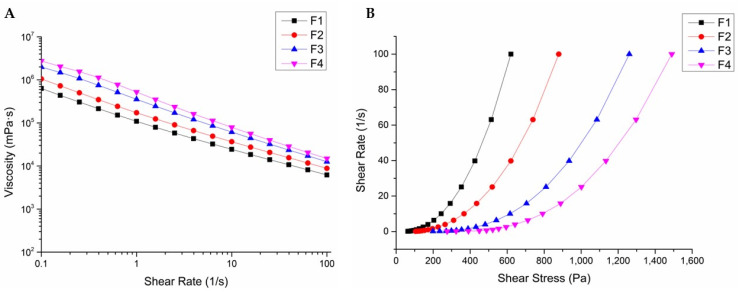
Rheology assessment: (**A**) variation in viscosity curves of emulsion gels with pectin concentrations ranging from 5% to 8% (*w*/*w*); (**B**) rheological curves for shear stress versus shear rate of emulsion gels.

The viscosity of each emulsion formulation is presented in Figure 2A. As the shear rate applied to the emulsions increased, their viscosity decreased, demonstrating shear-thinning behavior. At a shear rate (1/s) of 1, the viscosities of the formulations (F1, F2, F3, and F4) were 109,240 mPa·s, 172,530 mPa·s, 353,730 mPa·s, and 521,890 mPa·s, respectively. These results indicate that higher pectin concentrations result in higher viscosities [30,31]. Notably, the viscosity of formulation F4, which contained the highest concentration of pectin, was approximately 4.7 times greater than that of formulation F1.

The relationship between shear rate and shear stress (Figure 2B) demonstrated the pseudoplastic flow behavior of the emulsion gel. This behavior not only ensures ease of injection but also facilitates the administration of emulsion gels to tissue surfaces [32]. Once the gel has been applied, the viscosity of the emulsion is expected to recover, enabling it to adhere to the tissue for an extended period. This characteristic is critical for maintaining functionality in situ and enhancing therapeutic efficacy.

The pseudoplastic behavior of the emulsion gel plays a critical role in allowing the product to meet these criteria. This shear-thinning property allows the gel to flow easily under applied force (e.g., during injection) but recover its viscosity once the force is removed, ensuring stability and retention at the surgical site. Therefore, the pseudoplastic nature of the formulation enhances its applicability in surgical settings by providing both ease of administration and prevention of sustained adhesion [33].

### 3.2. In Vitro Mucoadhesion Assessment

**Figure 3:**
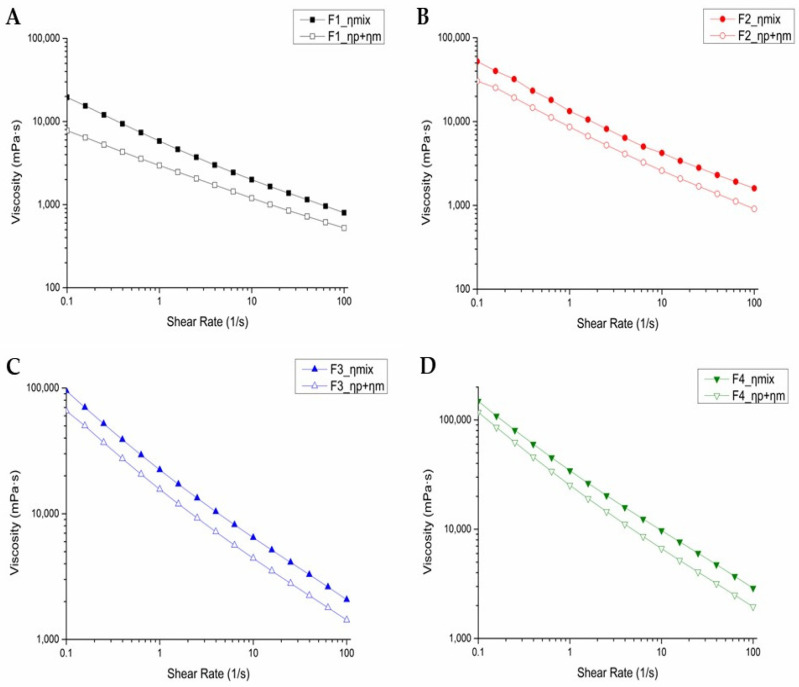
Viscosity-versus-shear-rate curves for the sum of mucin suspension and emulsion gel (open symbols) and the mixture of mucin with emulsion gel (filled symbols): (**A**) F1 (pectin 5%), (**B**) F2 (pectin 6%), (**C**) F3 (pectin 7%), and (**D**) F4 (pectin 8%).

The mucoadhesive properties of emulsion gels were assessed to evaluate the role of pectin. Pectin contains ester and carboxylic acid groups that facilitate hydrogen-bonding interactions [11]. For this reason, using pectin supports stronger interactions between anti-adhesion agents and mucins in the mucosa. Figure 3 shows the relationship between the mixed viscosity (F_ηmix_) of the formulation and the mucilage and between the sum of the individual viscosities of the formulation (F_η_) and the mucilage (η). All formulations showed an increase in F_ηmix_, indicating enhanced viscosity resulted from the interaction between the formulation and the mucin. The shear rate was fixed at 1 s^−1^ throughout the measurements to ensure consistency in viscosity evaluation. Under this condition, the Δη values were observed to be 2856 mPa·s for F1, 4679 mPa·s for F2, 6555 mPa·s for F3, and 9009 mPa·s for F4. This suggests that an increased amount of pectin facilitates strong interactions between the formulations and the mucin by enhancing viscosity. Formulation F4, which contained the highest pectin content, exhibited the greatest mucoadhesive strength. F4 could effectively adhere to the mucosal surface and remained on the tissue for a prolonged period. Such behavior is critical for sustained drug release and prolonged retention at the site of action. These results underscore the role of pectin concentration in enhancing mucoadhesive properties, further supporting its utility in anti-adhesion emulsion gels.

### 3.3. Particle-Size and Zeta-Potential Measurements

The particle size, zeta potential, and polydispersity index (PI) of all prepared emulsion gels were measured (Table 4). The results for particle size showed no significant differences, with PI values ranging between 0.1 and 0.2. The reason for this is that the same type of surfactant was used in the same amount in all formulations. A zeta potential of approximately ±30 mV is known to generate sufficient electrostatic repulsion to prevent particle agglomeration [34]. In this study, the zeta potentials of all formulations ranged from −32 mV to −40 mV, suggesting that particle aggregation is effectively inhibited under these conditions.

### 3.4. In Vitro Assessment of Emulsion-Gel Degradation 

**Figure 4:**
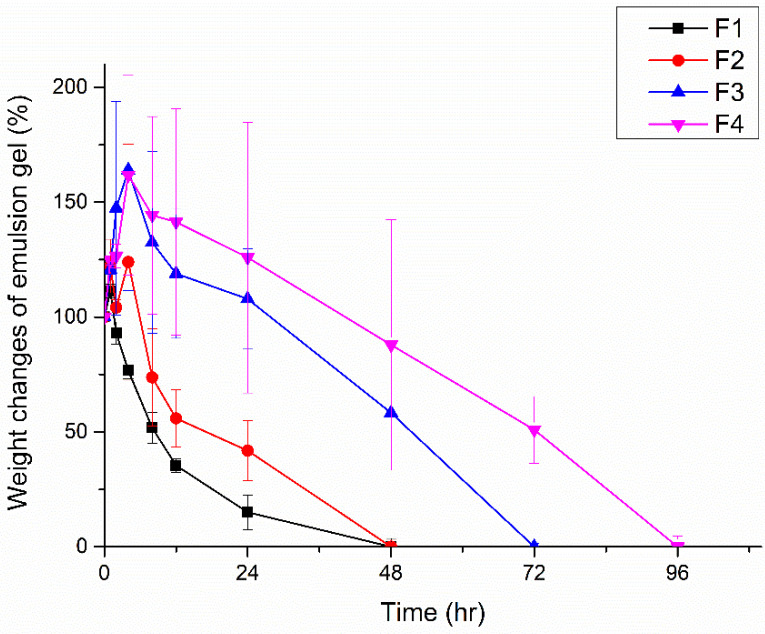
Weight changes of F1, F2, F3, and F4 in PBS buffer (pH 7.4) (n = 3).

Figure 4 depicts the degradation of the formulations in an environment similar to the abdominal cavity. A slower degradation rate allows the emulsion to function more effectively as a physical barrier by allowing it to maintain its shape within the abdominal cavity [35]. The results indicate that a higher pectin content in the formulation leads to a slower degradation rate. Specifically, F1, which had the lowest pectin content, completely degraded within one day, whereas F4, with the highest pectin content, took four days to fully degrade. The initial increase in weight is attributed to the water-absorption capacity of pectin, which can aid wound healing by allowing the gel to absorb exudate from the wound site.

### 3.5. In Vitro Assessment of CEL Release 

**Figure 5:**
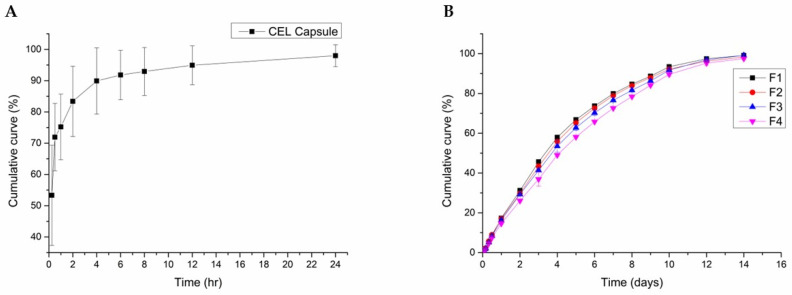
Release profiles of CEL-loaded emulsion gels (n = 3): (**A**) celecoxib capsule and (**B**) emulsion gels.

The aim of this study was to evaluate whether a pectin-based emulsion gel could be utilized as a drug-delivery system, specifically by examining the release profile of CEL. To achieve this, in vitro release studies were performed in PBS (pH 7.4, 37 °C). As a comparison group, commercially available CEL capsules were used, with their encapsulated powder extracted for testing (Figure 5). The release of CEL from the pectin-based emulsion gels was sustained for 14 days, with the CEL exhibiting a similar overall release pattern across formulations. The formulation with the higher pectin content correlated with the most sustained release. F1, which contained the lowest pectin concentration, released approximately 80% of the CEL by day 7 and 100% release by day 14, for the fastest release rate. In contrast, F4, which had the highest pectin content, released 80% of the CEL by day 9, demonstrating the slowest release rate. The commercial CEL formulation exhibited a burst-release profile, with 80% of the CEL released within 2 h and complete release achieved within 24 h (Figure 5A). Although this is useful for immediate pain relief, frequent administration is required, which leads to potential toxicity risks and patient inconvenience [36,37]. In contrast, the developed emulsion gels demonstrated a sustained-release pattern (Figure 5B). This could reduce the need for frequent oral administration, minimizing potential systemic toxicity and providing local postoperative pain relief at the operative site [36].

### 3.6. In Vivo Evaluation of Intraperitoneal Anti-Adhesion Effects

**Figure 6:**
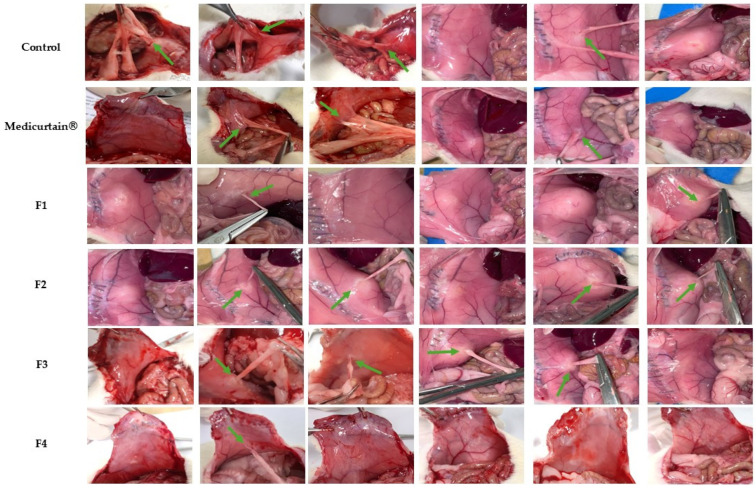
In vivo evaluation of anti-adhesion effects (n = 6). Green arrows indicate adhesion at the abrasion site.

**Figure 7:**
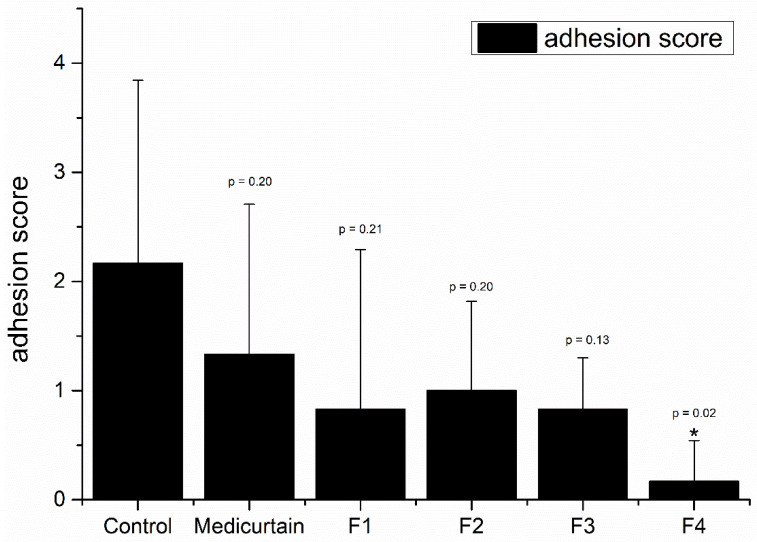
In vivo anti-adhesion performance with observation after 10 days (n = 6). * *p* < 0.05 (vs. control).

To evaluate their intraperitoneal anti-adhesion effects, the formulations were administered to laparotomy models established in 8-week-old Sprague−Dawley (SD) rats with abraded peritoneums. To assess the efficacy of adhesion prevention, we assigned the rats to a no-treatment control group and a group treated with Medicurtain^®^, which is a commercially available anti-adhesion agent. Additionally, emulsion-gel formulations (F1–F4) with varying pectin contents were evaluated for their adhesion scores. In each group, a laparotomy was performed and the left peritoneum was intentionally abraded using sandpaper before the formulations were applied. Laparotomy was repeated 10 days post-surgery (Figure 6). The adhesion-scoring criteria are presented in Figure 7. The control group had an average adhesion score of 2.17, while the Medicurtain^®^ group scored 1.33 on average. Among the experimental groups, the average scores were as follows: F1, 0.83; F2, 1.00; F3, 0.83; and F4, 0.17 (Figure 7). F4 demonstrated the most effective adhesion prevention among all test formulations. Upon visual inspection, the wound sites appeared well-healed, with no abnormal findings observed.

### 3.7. Histopathological Analysis of Intraperitoneal Tissue

**Figure 8:**
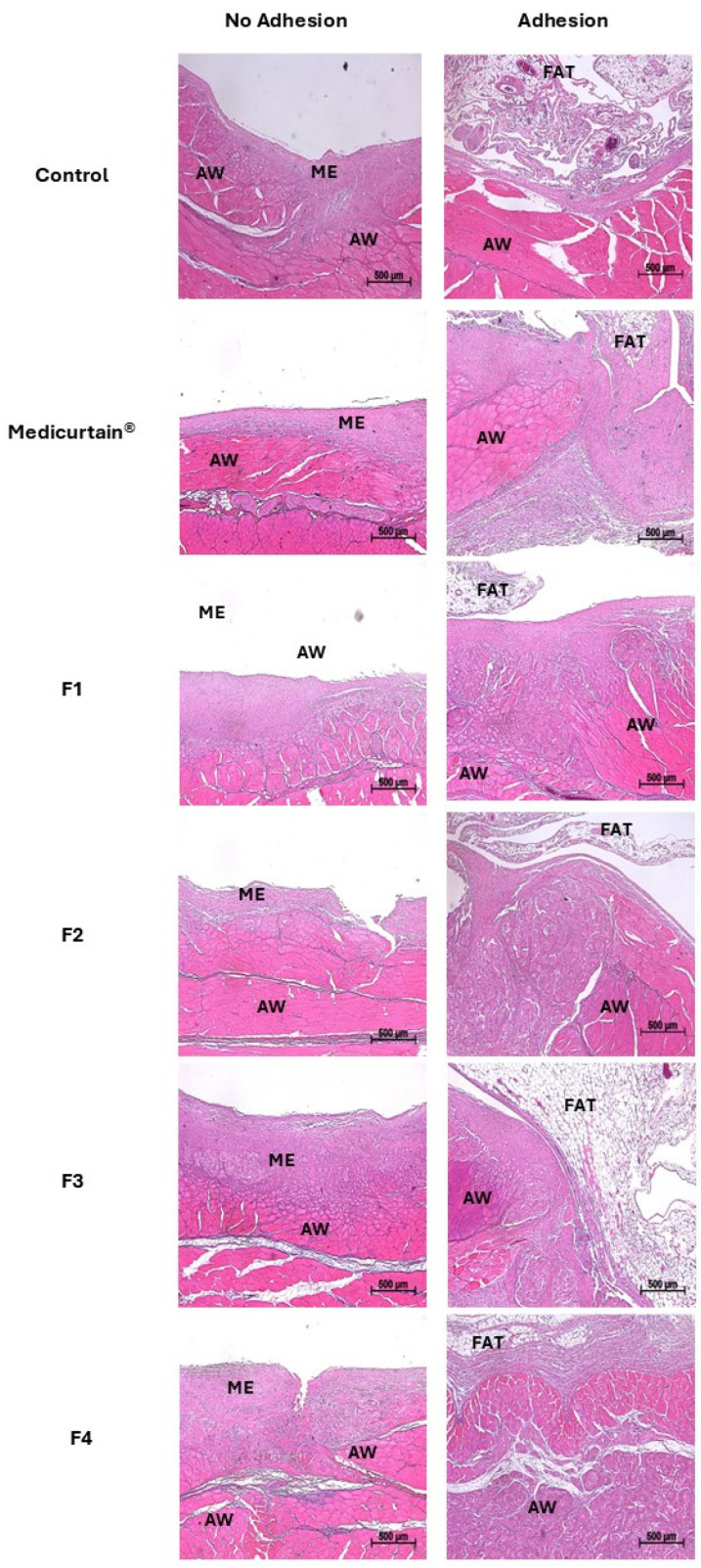
Representative H&E-stained images of adhesion and non-adhesion tissues collected from each group, analyzed 10 days post-surgery. AW, abdominal wall; ME, mesothelial band (scale bar: 500 μm).

To induce adhesion formation, the abdominal wall was subjected to approximately 200 abrasions using sandpaper before formulations were applied. After 10 days, adhesion formation was assessed and tissue samples were collected from both rats with adhesion formation and those without adhesion formation in each experimental group (Figure 8). Histological analysis revealed that in rats without adhesion formation, the injured peritoneum was fully covered by a continuous layer of neomesothelial cells. In contrast, in rats with adhesion formation, fibrotic adhesions were observed between the damaged peritoneum and adjacent fat tissue, with the formation of connective tissue bridging these structures. Furthermore, in F4, which exhibited the lowest adhesion score, less extensive adhesion between the peritoneum and fat tissue was observed compared to other groups.

### 3.8. In Vivo PK

**Figure 9:**
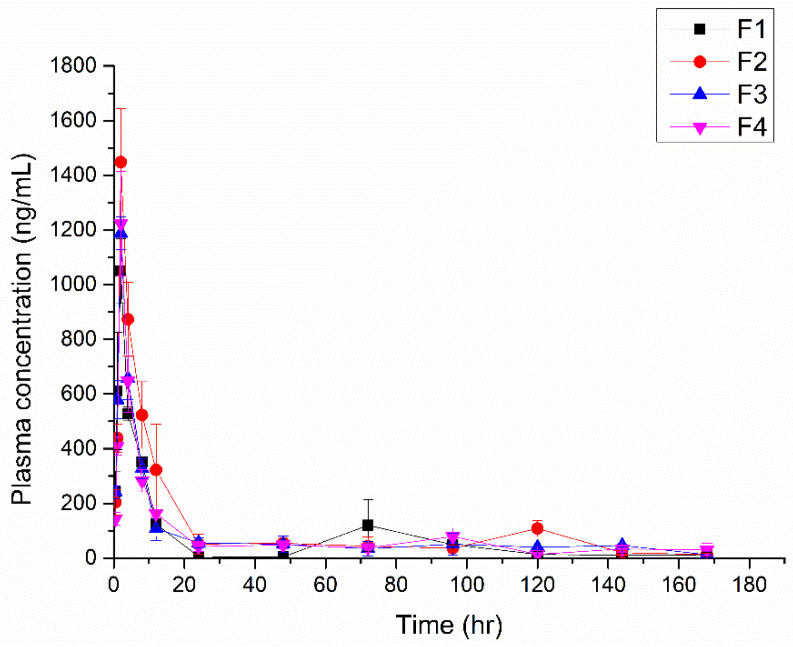
Plasma-concentration-versus-time profiles of CEL-loaded emulsions in SD rats (n = 3).

This study was conducted to evaluate the plasma PK of CEL. The release of CEL from the emulsion gel into the bloodstream was observed in an SD rat model. The sample was administered after the abdominal cavity had been abraded 200 times with sandpaper to simulate surgical conditions. The blood concentration of CEL over time following the application of formulations F1, F2, F3, and F4 was also measured (Figure 9). The PK parameters (C_max_, T_max_, and AUC) are presented in Table 5. The T_max_ of all formulations was 2 h, and most blood concentrations declined within 24 h. These results suggest that the formulations could effectively release CEL in the postoperative phase, potentially reducing pain and inflammation immediately after surgery. Moreover, the findings highlight the potential of CEL-loaded emulsion gels as a localized drug-delivery system that could be used to enhance pain management following surgical procedures.

## 4. Conclusions

In this study, we developed an anti-adhesion emulsion gel using safe, non-toxic, and biocompatible soybean oil and pectin. In the rheological tests, all formulations exhibited pseudoplastic flow, making them convenient for application to tissue surfaces. The in vitro mucoadhesion study and in vitro degradation test demonstrated that the F4 formulation, which had the highest pectin content, remained in the abdominal cavity for the longest duration and adhered to the surface of the tissue to which it was applied for an extended period. Consequently, in vivo intraperitoneal anti-adhesion experiments visually confirmed that the F4 group showed evidence of superior anti-adhesion effects compared to both the control group and the Medicurtain^®^ group. Furthermore, CEL was incorporated into the emulsion to alleviate postoperative pain. The in vitro CEL-release study indicated that drug release was sustained for 14 days. Although the overall release patterns were similar among formulations, those with higher pectin content exhibited a relatively slower release rate. In vivo PK analysis confirmed that the T_max_ of all formulations was 2 h, indicating that the drug was rapidly absorbed at the surgical site and subsequently transferred into systemic circulation. No significant differences in the release patterns among the formulations were observed. Therefore, we successfully designed an emulsion capable of rapidly reducing postoperative pain while effectively preventing adhesions. However, differences between in vitro and in vivo drug-release rates are likely due to the low molecular cut-off of the membrane used in the in vitro-release study. To obtain more accurate results, a different membrane should be considered for future studies.

## Figures and Tables

**Table 1:** Formulation of emulsion gels (unit: gram scale).

	Celecoxib	PEG 400	Pectin	Water	P 407	Montanox^®^ 80	Soybean Oil
F1	0.03	2.0	0.5	5.04	0.01	0.05	2.4
F2	0.03	2.0	0.6	4.94	0.01	0.05	2.4
F3	0.03	2.0	0.7	4.84	0.01	0.05	2.4
F4	0.03	2.0	0.8	4.74	0.01	0.05	2.4

**Table 2:** Adhesion severity grading scale.

Grade	Descriptions
0	No adhesions
1	Tiny, filmy adhesions easy to separate
2	Dense adhesion that require tension to divide
3	Dense adhesions that require division by scissors
4	Other intraabdominal organs involved, with conglomerate formed

**Table 3:** Storage modulus (G′), loss modulus (G″), loss factor (tan δ), and angle (°) of loss factor (δ) at an angular frequency of 10 rad/s.

Sample	Storage Modulus (Pa)	Loss Modulus (Pa)	Loss Factor (tan δ)	δ (°)
F1	957.4	304.18	0.318	17.6
F2	1761.9	528.11	0.300	16.7
F3	6401.9	1153.8	0.180	10.2
F4	7444.2	1285.3	0.173	9.8

**Table 4:** Particle size, polydispersity index (PI), and zeta potential of each emulsion gel.

Sample	Particle Size (nm)	Size PDI	Zeta-Potential (mV)
F1	1121 ± 167.7	0.117 ± 0.08	−39.40 ± 0.96
F2	1041 ± 162.4	0.193 ± 0.05	−34.45 ± 0.74
F3	933 ± 57.7	0.119 ± 0.06	−32.35 ± 0.48
F4	987 ± 77.2	0.156 ± 0.03	−32.39 ± 1.33

**Table 5:** In vivo pharmacokinetic parameters of SD rats (n = 3).

Sample	C_max_ (ng/mL)	T_max_ (h)	AUC_(0→168 h)_ (ng·h/mL)
F1	1048.9 ± 145.3	2 ± 0	11,095.2 ± 2679.3
F2	1448.1 ± 239	2 ± 0	17,230.3 ± 4863.2
F3	1187.7 ± 72.9	2 ± 0	12,941.2 ± 3715.7
F4	1223.0 ± 234.1	2 ± 0	12,743.6 ± 740.7

## Data Availability

Data are available upon request.
